# Uncertainty-Weighted Time Averaging of Mercury Vapour Concentrations in Ambient Air: Application to Measurements in the United Kingdom

**DOI:** 10.1100/tsw.2011.37

**Published:** 2011-02-03

**Authors:** Richard J. C. Brown, Dharsheni Muhunthan

**Affiliations:** Analytical Science Division, National Physical Laboratory, Teddington, Middlesex, UK

**Keywords:** mercury vapour, time averaging, uncertainty, ambient air, United Kingdom

## Abstract

Uncertainty-weighted time averaging of total gaseous mercury concentrations in ambient air, with associated robust uncertainties, has been performed for concentrations measured by the U.K. Heavy Metals Monitoring Network between 2007 and 2009. The results have been compared with averages produced using standard time-averaging methods with a view to investigating the properties of the new method and whether it represents an improvement over current practice.

## INTRODUCTION

Mercury remains an important environmental pollutant because of its toxicity and persistence in terrestrial and aquatic biosystems. The deposition of mercury-containing compounds onto land and water is a major concern. As those deposited portions of mercury may eventually enter the food chain, its presence in air where the general population can readily inhale it is also of importance[1]. Ambient air legislation covering ambient mercury vapour mainly targets the allowable levels of gaseous elemental mercury, not only for assessment of population exposure, but also to gauge long-term concentration trends and the effectiveness of abatement strategies from stationary sources and industrial processes. Gaseous elemental mercury is measured because it is the most abundant, if not the most toxic, form of mercury present in the atmosphere[2]. Moreover, it is simpler to measure accurately and traceably than other forms of mercury in the atmosphere. As a result of legislative requirements to measure “total gaseous mercury”[3] (TGM – taken to mean gaseous elemental mercury under most circumstances, since contributions to mercury in the atmosphere from reactive gaseous mercury and particulate-bound mercury are extremely small under most conditions[4]), national air quality networks now include monitoring stations to measure TGM concentrations at background locations and close to point sources[5], using internationally agreed standard methods[6,7]. International cooperation in this area is particularly important since mercury vapour is a global pollutant contributing to transboundary air pollution issues[8].

Accurate assessment of ambient concentration levels over prolonged periods requires accurate and traceable individual measurements and confidence in the methodology used to produce average values from a time series of measurements[9]. European policy refers to international standards for performing these assessments. These standards identify the simple mean as the best estimate of an average from a time series and an uncertainty in this average that is related to the uncertainties of the individual measurements in this time series[10]. This approach has two drawbacks: (1) it does not make use of the uncertainty in individual measurements to produce the time-averaged value and (2) a few input values with very large uncertainties may have a disproportionate influence on the uncertainty of the final time-averaged value, possibly meaning that it does not meet the data quality objectives of the legislation that the measurements are intended to address.

We recently proposed a novel method of performing time averaging, which used information about the uncertainty of individual measurement results to produce an uncertainty-weighted average of a time series of measurements and a robust uncertainty estimate based on a least median squares assessment of how uncertainty is related to the concentration level being measured[11]. Moreover, this method has the advantage of using data gained from a much larger set of measurements to produce the statistical basis for the time-averaged value and its uncertainty, not just the data that are part of the time average under consideration.

This method is now applied to a novel investigation of the time averaging of measurements of TGM in the U.K. as part of the U.K. Heavy Metals Monitoring Network[12]. It is proposed that the technique is more suitable for use with TGM concentration data than for the previous demonstration on exemplar particulate-bound metals data, since relative uncertainties of mercury vapour measurements vary considerably more with concentration. This is as a result of the larger and more variable uncertainties in sample volume, analytical recovery, analytical drift, and repeatability, as compared to the analysis of particulate-bound metals. This paper compares the annual average values obtained at nine sites around the U.K. that have measured TGM during 2007, 2008, and 2009, using the traditional time-averaging procedure and the novel uncertainty-weighted method, and examines in detail the differences in the two datasets produced. There is currently very little work in the literature, to the best of the authors' knowledge, that examines different methods for evaluating annual average values and their uncertainties for air quality metrics[13,14].

## EXPERIMENT

The U.K. Heavy Metals Monitoring Network helps to discharge the U.K.'s obligations under the European Air Quality Directives relating to particulate-bound metals and TGM[15]. The Network usually maintains 13 TGM monitoring sites, but owing to a reorganisation of Network sites during 2007 and 2008, to ensure compliance with the requirements of the European legislation, the number of sites producing TGM concentration data continuously from 2007 to 2009 was only nine. Data from all nine of these sites have been used in this study. Measurements of TGM are currently carried out by sampling ambient air for a period of between 1 and 4 weeks (depending on the expected ambient concentration levels[16]) onto an adsorption tube (containing gold-coated silica as the sorbent material) at each monitoring site using a calibrated low-volume pump sampling at approximately 300 ml/min. A filter is attached to the front of the sampling line to prevent particulates reaching the adsorption tube. The adsorption tube is then sent to the National Physical Laboratory for analysis by atomic fluorescence spectrometry (PS Analytical Ltd, 10.525 Sir Galahad). This procedure has been described in detail elsewhere[17] and so only the main points are reproduced below.

The spectrometer is calibrated using known volumes of mercury-saturated air removed using a calibrated syringe from the so-called "bell-jar" calibration apparatus[18]. Prior to a series of measurements, the linearity of the instrument is confirmed using a series of five injections across the measurement range and the recovery of mercury from an adsorption tube dosed with a known mass of mercury is measured. Once linearity is established and the recovery is within allowable methods parameters (between 90 and 105%), the mercury content of the adsorption tubes is determined relative to an additional one-point calibration. This calibration injection is made prior to the measurement of each adsorption tube and tailored to a level close to the mass expected to be present on the tube[19]. Because mercury vapour concentrations in air show relatively little variability over time, and the drift of the analytical instrument is a greater contribution to measurement uncertainty than its nonlinearity, this is the most efficient and accurate way to make measurements. Uncertainties are calculated for the entire measurement using a full GUM[20] approach, which has been recently documented[17]. Contributions to uncertainty from lost time coverage are extremely small for the datasets examined and so have been neglected.

## RESULTS AND DISCUSSION

Expanded uncertainties for the measurement described are of the order of 17%[17], but may vary significantly owing to variability in the uncertainty of several key input parameters:

- Sample volume. Flow rates can often vary over time, making final sample volumes difficult to determine accurately.
- Recovery of mercury from adsorption tubes. The recovery of the tube used for the quality-assurance step prior to measurement may vary, and this will impact on the uncertainty of all measurements made until the next recovery test.
- Calibration. The uncertainty of the one-point calibration depends on the size of the injected mass and, critically, the closeness of this mass to that determined on the adsorption tube. Unexpectedly large differences may result in large uncertainties.
- Blank level. Each adsorption tube has a blank level, thought to be as a result of deeply adsorbed mercury and interfering compounds[17]. The higher this blank, the higher the uncertainty in the blank correction.

Variation in these parameters causes larger variations in overall relative measurement uncertainties than is usually observed with particulate-bound metals on the U.K. Heavy Metals Monitoring Network[11]. We used all measurement values and their uncertainties determined over all nine sites during 2007, 2008, and 2009 to determine the expected relationship between TGM concentration and expanded uncertainty by performing a robust least median squares regression[21,22], which minimises the median squared ordinate residual, yielding a relationship of the form $u = mx + c$. (This approach is highly robust and not influenced even by significant numbers of outliers.) The result of this analysis for the TGM data referred to above is shown in Fig. 1 – yielding a gradient of 0.174 and an intercept of 0.001 ng $m^{-3}$. As can be seen, the majority of the 684 measurements are close to the regression relationship determined. However, there are also a substantial number of significant outliers.

This relationship is now used to determine a weighting parameter, $w$, for each measurement value, related to the deviation of individual points from $u = mx + c$, and is given by:

$$w_i = \frac{u_i}{mx_i + c} \tag{1}$$

where $w_i$ is the weighting parameter determined for a measurement $i$ of value $x_i$ with an uncertainty of $u_i$. The weighting parameters determined for the set of results shown in Fig. 1 are displayed in Fig. 2.

An uncertainty-weighted average for a subset of these data representing a given time series (e.g., the 2007 annual average concentration in Cardiff), $\bar{x}_T$, may then be obtained thus:

$$\bar{x}_T = \frac{\sum_{i=1}^{N} x_i / w_i^2}{\sum_{i=1}^{N} 1 / w_i^2} \tag{2}$$

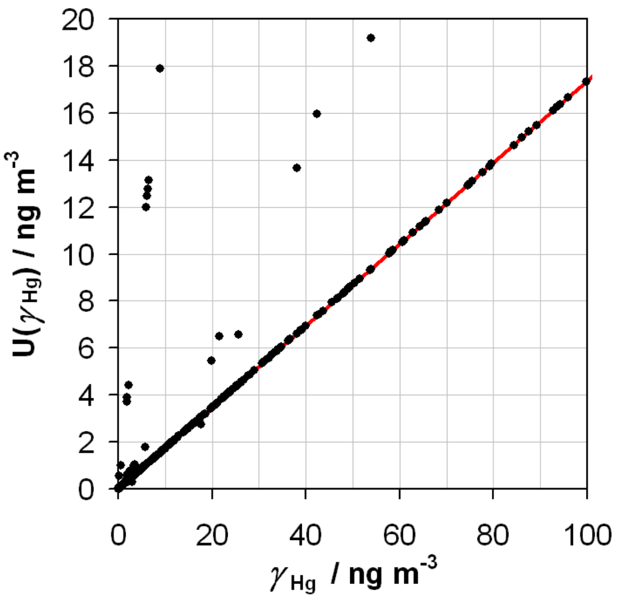

**FIGURE 1**. The expanded uncertainty, U($\gamma_{Hg}$), as a function of TGM concentration, $\gamma_{Hg}$, calculated for 684 measurements made at nine monitoring sites on the U.K. Heavy Metals Monitoring Network from 2007 to 2009 inclusive. The red line indicates the least median squares best fit to the data, of the form $u = mx + c$.

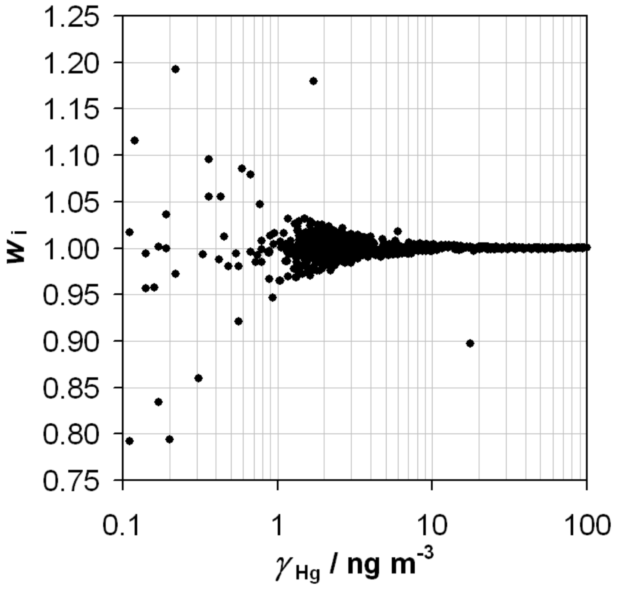

**FIGURE 2**. The weighting parameters, $w_i$, determined using Eq. 1, as a function TGM concentration, $\gamma_{Hg}$, for the 684 measurements made at nine monitoring sites on the U.K. Heavy Metals Monitoring Network from 2007 to 2009 inclusive. Seventeen values are off-scale at the top of the graph for $1.25 < w_i < 3.50$. There is one value off-scale at the bottom of the graph at $w_i = 0.60$.

The uncertainty of this weighted average is then given simply by the position of $\bar{x}_T$ on the least median squares best-fit line, thus:

$$\bar{u}_T = m\bar{x}_T + c \tag{3}$$

The annual average concentrations for 2007, 2008, and 2009, at each of the nine Network sites has been determined using the method described above and also using the ISO 11222 approach (referred to here as the simple mean approach), where the annual average is the simple mean and the uncertainty is related to the individual uncertainties in the data comprising the time series. The results of this analysis are shown in Fig. 3A and B.

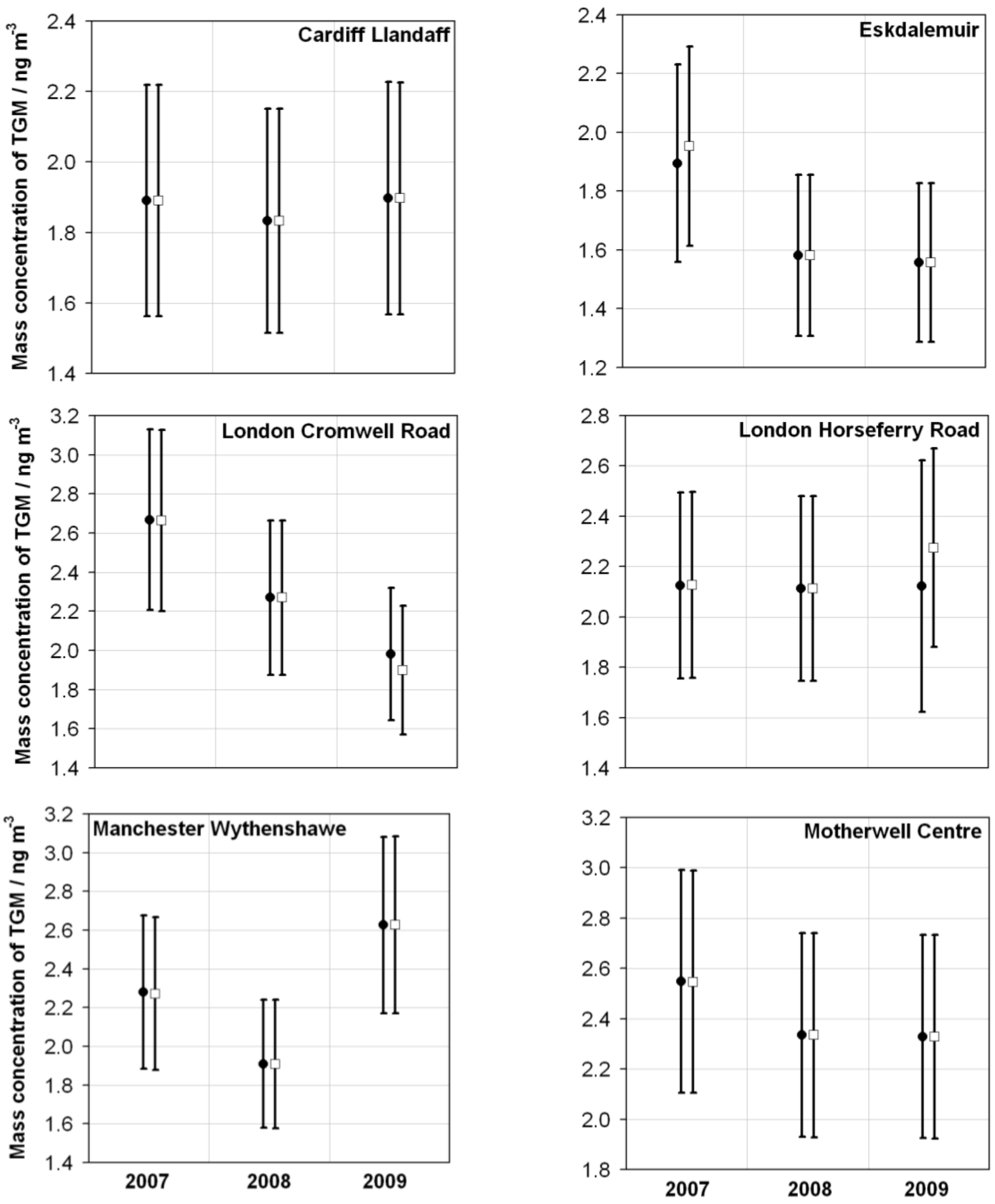

**FIGURE 3A.** The annual average mass concentration of TGM from 2007 to 2009 inclusive, and the expanded uncertainty in this average value, calculated using the simple mean approach (left-hand bars, filled circles) and the uncertainty-weighted mean method (right-hand bars, empty squares) at six monitoring sites on the U.K. Heavy Metals Monitoring Network (as indicated at the top of each chart).

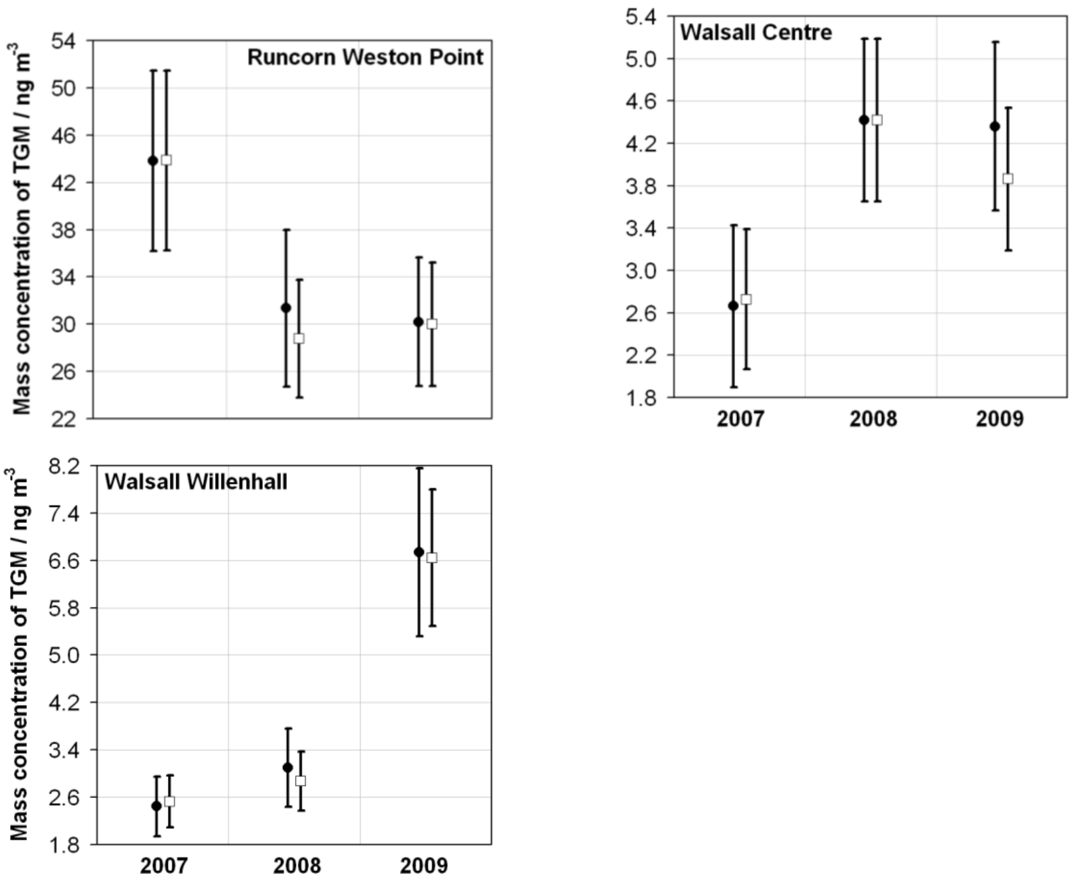

**FIGURE 3B.** The annual average mass concentration of TGM from 2007 to 2009 inclusive, and the expanded uncertainty in this average value, calculated using the simple mean approach (left-hand bars, filled circles) and the uncertainty-weighted mean method (right-hand bars, empty squares) at three monitoring sites on the U.K. Heavy Metals Monitoring Network (as indicated at the top of each chart).

As can be seen, a large number of annual average values and relative uncertainties have remained within 0.5 % (relative) of the original values: 17 out of the 27 pairs of values displayed. However, 12 pairs of values have shown a significant change in either the annual average value or the relative uncertainty in this average as a result of using the uncertainty-weighted mean (UWM) approach as opposed to the simple mean (SM) technique. As can be seen in Fig. 4, where the observed changes for each parameter have been ranked, there has been a significant increase (>0.5% relative) in four annual average values and one relative annual average uncertainty value, and a significant decrease (>0.5% relative) in six annual average values and nine relative annual average uncertainty values. However, all pairs of values produced using the UWM and SM methods are mutually consistent at the 95% confidence level.

The cause of significant decreases in annual average values is generally the presence of very large values within the dataset under consideration, with much-larger-than-expected uncertainties, e.g., Walsall Centre in 2009. However, this may also occur when a number of low values are present with a lower-than-expected uncertainty. Conversely, increases in annual average values occur usually when there are a large number of low values with unexpectedly high uncertainties within the dataset, e.g., London Horseferry Road, and possibly, but more rarely, when a few high values with lower-than-expected uncertainties are present. Because, as Fig. 2 shows, there is a relatively even occurrence of weightings higher and lower than one (except for extreme outliers – almost always with very high uncertainties), the number of annual average values showing a significant decrease is similar to the number showing a significant increase.

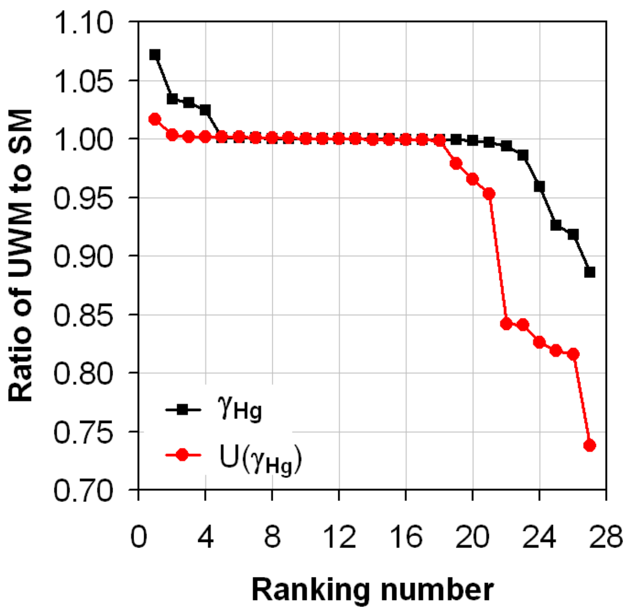

**FIGURE 4.** The ratio of the TGM concentrations, $\gamma_{\mathrm{Hg}}$ (black squares), and expanded uncertainties, U($\gamma_{\mathrm{Hg}}$) (red circles), calculated using the UWM method to those calculated using the SM approach, and ranked in descending order independently for each parameter. The data represent annual values at nine U.K. Heavy Metals Monitoring Network sites for 2007 to 2009 inclusive.

The situation is different for the uncertainty in the annual average. The value produced by the UWM method is robust and dependent only on the annual average value and the $u = mx + c$ relationship determined from the larger dataset. The value produced by the SM method recommended in ISO 11222[10] is dependent on the input uncertainties in the subset of data used to create the annual average in question. In this case, uncertainties that are larger than expected have a much larger leverage on the calculated value than uncertainties that are smaller than expected; this is approximately a squared relationship. Therefore, using the SM method, only a few larger-than-expected uncertainties (roughly speaking, in excess of the square root of the number of uncertainties that are smaller than expected) are required to produce a larger uncertainty in the annual average than obtained using the UWM method. Conversely, a much larger number of smaller uncertainties are required (roughly speaking, in excess of the square of the number of uncertainties that are larger than expected) to produce an uncertainty in the annual average that is smaller than that obtained using the UWM method. Since the distribution of weightings, as shown in Fig. 2, is relatively symmetrical, about 1, it is much more likely that uncertainties in the annual mean using the UWM method will be less than those produced using the SM method. The relationship between the changes in the annual average value and the uncertainty in this value for each pair of results is shown in Fig. 5.

The absence of a clear relationship between the two sets of values indicates the relatively independent treatment of concentration and uncertainty data by the UWM method. However, the spread of data reinforces the observation that the UWM method generally decreases uncertainty, but causes little change overall in annual average values, with a large number of values remaining unchanged. Indeed, the overall change averaged over the 27 values considered was –0.7% (relative) for the annual average and –4.4% (relative) for the uncertainty in the annual average. Finally, Fig. 6 examines the relationship between the change observed in the values obtained when using the UWM method as opposed to the SM technique, and the average TGM concentration at each site.

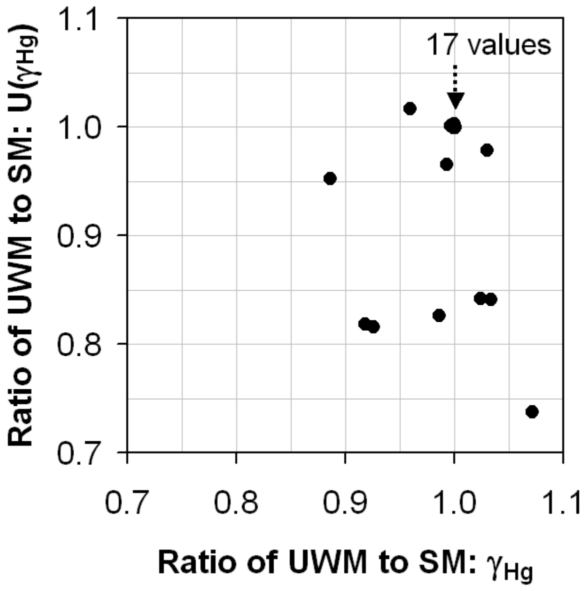

**FIGURE 5.** The ratio of the TGM concentrations, $\gamma_{Hg}$, calculated using the UWM method to those calculated using the SM approach, against the ratio of the expanded uncertainties in those TGM values, U($\gamma_{Hg}$), calculated using the UWM method to those calculated using the SM approach. The data represent annual values at nine U.K. Heavy Metals Monitoring Network sites for 2007 to 2009 inclusive.

There is some evidence that as the average TGM concentration increases, the decrease observed in both the annual average and the expanded uncertainty in this annual average as a result of using the UWM technique becomes larger. However, these relationships are very weak (squared correlation coefficients, $R^2 < 0.5$), and are probably a function of the nature of the datasets under consideration (especially the outlying set at high concentration from Runcorn Weston Point, which has a large effect on any trend) and not a fundamental property of the technique itself.

## CONCLUSION

A technique for producing uncertainty-weighted means for time series of air quality data has been applied to measurements of TGM in the U.K. The technique is considerably more suited to TGM measurements than those of metals in particulate matter, for which the technique was originally proposed. This is because TGM measurements exhibit far greater variations in relative uncertainty across the concentration domain. Of the 27 annual averages to which the UWM technique were applied, 10 showed significant differences when compared to the results obtained using the SM method. Some of the properties of the technique have also been investigated. It has been found that variations in the annual mean value are generally small and are roughly distributed around the original value, whereas variations in uncertainty of the annual average are much larger and generally produce lower values as compared to the SM method.

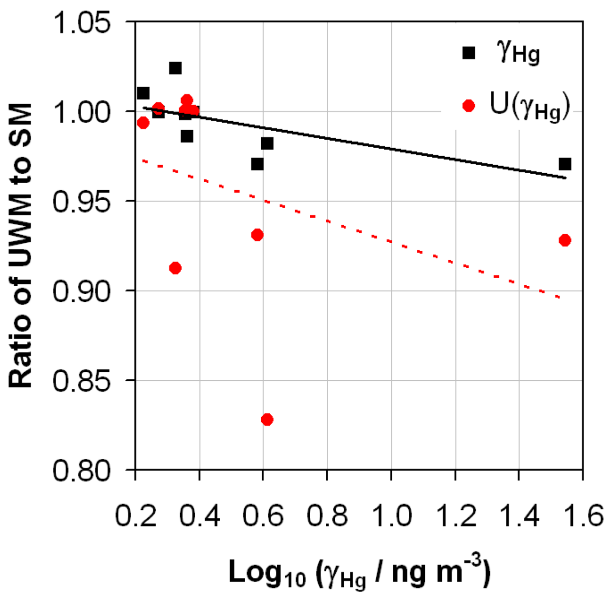

**FIGURE 6**. The ratio of the TGM concentrations, $\gamma_{Hg}$ (black squares), and expanded uncertainties, U($\gamma_{Hg}$) (red circles), calculated using the UWM method to those calculated using the SM approach, as a function of TGM concentration. The data represent 3-year averages (from 2007 to 2009 inclusive) at nine U.K. Heavy Metals Monitoring Network sites. Lines of best fit are shown for TGM concentrations (solid, black) and expanded uncertainties (dashed, red).

It is proposed that the method presented is a possible improvement on existing methodologies for calculating annual means and uncertainties in annual means for time averages. We propose a number of reasons:

- The robust assessment of uncertainty has the effect that values are not disproportionately affected by a small number of values with very high uncertainties. This provides a more sensible assessment of the uncertainty in the annual average and means that compliance with legislative limits for maximum allowable measurement uncertainty is more likely to be met.
- The UWM method makes use of the individual uncertainty statements provided for each measurement, thereby making a more intelligent assessment of the annual average value with, in most cases, a justifiably higher confidence associated with the value.
- The dataset from which the robust uncertainty and the weighting parameters is calculated is much larger and, therefore, more representative of the overall measurement procedure than the use of parameters simply restricted to the dataset under consideration for the average being calculated.
- The relatively close agreement between the UWM and SM methods means that, if such an approach were ever adopted, step changes in air quality data produced would be unlikely.

However, the UWM method is dependent on rigorous and justifiable assessments of uncertainty for each measurement result, and the assumption that the robust relationship between TGM concentration and relative uncertainty does not change significantly during the measurement period under consideration. This may present a problem for measurements performed in a more routine, high-throughput manner. Only if these requirements were in place would the novel method presented here represent a possible improvement on the traditional method. When standard methodologies for such calculations are reviewed, it is proposed that this generic methodology be considered, especially for pollutants where there is significant variability in relative uncertainty with concentration and where individual statements of measurement uncertainty are produced.

## ACKNOWLEDGEMENTS

The U.K. Department for Business, Innovation, and Skills' funding of the National Measurement System Chemical & Biological Metrology Programme, and the U.K. Department for Environment, Food and Rural Affairs' funding of the National Physical Laboratory's operation and management of the U.K. Heavy Metals Monitoring Network, are both gratefully acknowledged.
